# Insights into the Feasibility and Acceptability of a Mobile Insulin Titration Application in Clinical Practice and Its Effects on Diabetes Outcomes

**DOI:** 10.17925/EE.2024.20.1.10

**Published:** 2024-02-29

**Authors:** Jodi S Krall, Jason M Ng, Neha Mehrotra, Kristine Ruppert, Linda M Siminerio

**Affiliations:** 1. University of Pittsburgh Medical Center, Pittsburgh, PA, USA; 2. niversity of Pittsburgh, Department of Medicine, Division of Endocrinology and Metabolism, Pittsburgh, PA, USA; 3. University of Pittsburgh School of Public Health, Department of Epidemiology, Pittsburgh, PA, USA

**Keywords:** Diabetes mellitus, diabetes self-management education and support, digital tools, glycated haemoglobin (HbA1c), insulin infusion systems, insulin technology, titration, type 2 diabetes mellitus

## Abstract

**Introduction**: Insulin therapy is most effective if patients learn how to properly adjust insulin to achieve glycaemic targets. There is a need for methods and tools that can assist these processes in clinical practice. The purpose of this feasibility study was to evaluate an approach to support insulin dose adjustment in individual patients using a mobile titration application (app). **Methods**: A cohort of adults (N=36) with type 2 diabetes with suboptimal glycaemia who were starting basal insulin self-titration were trained by a diabetes care and education specialist to use a mobile titration app to guide adjusting insulin doses. Glycaemia, diabetes distress and patient and provider satisfaction were assessed during the first 3 months after initiating basal insulin titration using the mobile app. **Results**: Mean haemoglobin type A1c (HbA1c) was significantly reduced by an average of 2.1 ± 2.2% from baseline to 3 months (p<0.001). Diabetes distress significantly decreased from baseline to follow-up with scores going down (or improving) across all scales. Both patients and providers reported high levels of satisfaction and positive experiences. **Conclusion**: The model offers a promising solution to streamline insulin dosage adjustments to achieve specific clinical and self-management goals with high expectations for long-term benefits and warrants further investigation.

Despite the increasing body of knowledge of treatment strategies for diabetes, many patients with type 2 diabetes mellitus (T2DM) are still in a persistent state of poor glycaemia.^1,2^ In clinical practice, achieving optimal glycaemic targets is challenging; the reasons are complex, as both patient-and healthcare provider (HCP)-related factors can play a significant role.^3,4^

When lifestyle and medication therapies are no longer effective in meeting treatment goals for patients with T2DM, insulin is prescribed and often started during a routine clinical visit or hospitalization.^5^ A number of challenges are associated with introducing insulin therapy, including clinical therapeutic inertia, unavailability or lack of self-management education, and patient-related fears referred to as psychological insulin resistance.^6^ Providers have described diabetes as being more difficult to treat than other chronic diseases as it requires more monitoring and medication adjustment.^7^ While efforts are underway to expand the pool of HCPs who can provide these services, such healthcare challenges may lead to delayed initiation, inadequate titration and failure to adhere to prescribed basal insulin dosing in patients who require basal insulin therapy.^8–11^ Insulin dosage adjustments are carried out at irregular intervals during busy clinical visits, resulting in patients often being under dosed thereby jeopardizing improved glycaemic outcomes.^12^

Insulin therapy is most effective if patients receive proper training and dosage titrations are done regularly and frequently. Diabetes self-management education and support (DSMES) provides the foundation to help people with diabetes navigate care activities and make complex daily decisions.^13^ There is also evidence that better quality of life and clinical outcomes can be achieved in those with chronic disease when allied HCPs make medication changes.^14,15^ Formerly referred to as diabetes educators, diabetes care and education specialists (DCES)^16^ are HCPs specifically trained to support the skills necessary to achieve glycaemic target goals and are considered experts in teaching patients diabetes medication-taking skills.^17^ DCESs have been reported to be able to effectively intensify treatment and reduce therapeutic inertia by providing expert advice related to the integration of diabetes technology into the clinical paradigm.^18–21^ The National Standards for the Medical Care of Diabetes and DSMES refer to the use of digital technology as a means to provide reach and real-time engagement in self-management, but note that additional assessment must be considered.^5,13^

Presently, HCPs typically rely on paper-based guides to help their patients titrate insulin doses. The paper-based tool is prepared as a worksheet with steps to determine the next insulin adjustment. A medical HCP determines an appropriate first dosage based on weight and blood glucose (BG) records. The patient is then expected to consult their HCP, who will teach them how to adjust the insulin dose based on their BG levels and give them a scale/algorithm to guide them on dose changes. The patient is expected to assume the 'lion's share' of the effort to titrate insulin using their 'worksheet' with some degree of teaching and unpredictable follow-up. Unfortunately, this process often sets the stage for initiation and titration delays. Patients have been described as experiencing potential delays of up to 3–6 months in dose adjustments.^22^ Consequently, there is a need for innovative tools and processes that can support patients in initiating and titrating their basal insulin and help to reduce the management burden for HCPs. In response, a mobile application (app) called 'My Dose Coach' (MDC) was developed for guiding titration of basal insulin therapy for people with T2DM and their HCPs. MDC is a smartphone app that provides a digital alternative to the manual titration model that is typically used for insulin dose modification. MDC provides similar functionality as a paper-based tool, while automatically calculating the median fasting blood glucose (FBG) value based on American Association of Clinical Endocrinology (AACE) guidelines and providing instruction on the next appropriate insulin dose according to a dose plan given by a medical HCP. The medical HCP defines an individualized longer-acting basal insulin titration plan that is used in MDC to give dose recommendations based on the patient's FBG and hypoglycaemic event data. In partnership, a DCES can receive and review the plan, provide DSMES on insulin therapy, and train and support the patient. In short, MDC offers a digital platform that represents a 'medical HCP-DCES' team-based solution for insulin titration and self-management education.

The primary goal of this feasibility study was to explore an approach that includes a DCES to oversee and support patient insulin dose adjustment using a digital titration app in real-world practice. The objectives were: (1) to assess glycaemia, diabetes distress and satisfaction in patients with T2DM during the first 3 months after initiating basal insulin titration with support of the digital titration app; and (2) to examine HCP satisfaction and acceptability of integration of the digital titration app into clinical workflow.

## Methods

This feasibility cohort study was approved by the University of Pittsburgh's Institutional Review Board. All procedures were followed in accordance with the responsible committee on human experimentation and with the Helsinki Declaration of 1964 and its later amendments. Written informed consent was obtained from study participants.

### Setting and study population

The study took place within the University of Pittsburgh Medical Center (UPMC) Health System in six diabetes clinics (one hub academic, one urban, two suburban, and three rural settings). Seven diabetes HCPs (six endocrinologists and one nurse practitioner) and six DCES (five nurses and one registered dietitian) from American Diabetes Association (ADA) recognized DSMES programmes, and their eligible patient populations, collaborated with the providers in their respective communities. Study participants included adult patients, aged 18 to 75 years, with T2DM and suboptimal glycaemia (defined as glycated haemoglobin A1c [HbA1c] of >7.5%) who were recommended to start basal insulin self-titration. Participants also had to be able to read and follow instructions in English, own a smart phone compatible with the digital app and be willing and able to install and use the MDC app.

### Recruitment and enrolment

A diabetes HCP identified patients during a routine clinic visit who met study eligibility criteria and were recommended to start basal insulin self-titration. Patients were given a brief introduction to the study, if they expressed interest, a full description of the study was provided. The patient was then referred for a baseline visit with a DCES for insulin therapy self-management education and MDC training. Recruitment ceased after 42 participants were enrolled into this feasibility study.

### Intervention

The DCES provided DSMES in keeping with National Standards for Diabetes Self-Management Education and Support.^13^ Sessions began with an assessment that included a review of clinical, behavioural and psychosocial needs and barriers, problem-solving skills and health literacy. Based on the assessment, the DCES provided education with specific attention to insulin therapy (e.g. a review of insulin actions, injection technique, when and where to administer insulin, prevention and treatment of hypoglycaemia, and a follow-up support process). In collaboration with the HCP, an individualized titration algorithm, including a dose plan and adjustment rules, was prepared and programmed into the MDC app. The DCES uploaded the insulin titration schedule into a provider MDC portal and sent an electronic link to the patient's smartphone via the text messaging feature to enable access to the app and initiate installation on their device. This was followed by a demonstration to the patient on the use of the MDC app to determine and guide dosing adjustments. Patients were told that the DCES and medical HCP would be available throughout the course of the study to respond to any question or concerns that the patient may have regarding insulin titration, insulin dosing, and the MDC app, specifically. In addition, patients were provided with insulin (glargine 100 IU/mL or 300 IU/mL, based on HCP recommendations) for the duration of their study participation.

### Endpoints

HbA1c served as a clinical endpoint to determine the impact of the process and MDC. HbA1c values were obtained from the patient's medical record. Change in FBG from baseline to 3 months, along with the percentage of patients who attained individualized titration target, time it took to attain titration target and occurrence of hypoglycaemic events (defined as FBG below the HCP-defined hypoglycaemia cutoff as per the titration plan) were captured from the MDC inherent data analytic system. Patients who logged three consecutive FBG measurements within their prescribed FBG target range (prespecified by the HCP during the care plan creation) were defined as reaching titration target.

Diabetes distress was evaluated at baseline and 3 months with the validated, self-reported, 17-item Diabetes Distress Scale (DDS17). The DDS17 assesses four dimensions of diabetes distress (emotional, regimen, interpersonal and physician) and has shown a consistent pattern of relationships with HbA1c, diabetes self-efficacy, diet and physical activity in multiple samples of patients with T2DM.^23,24^

Patient satisfaction was assessed using a study-specific survey (administered at 3 months) and the validated Diabetes Medication System Rating Questionnaire-Short Form (DMSRQ-SF) administered at baseline and 3 months. The diabetes medication system refers to the medication taken as well as the devices and supplies required to administer the medication. The DMSRQ-SF includes 20 items to assess convenience, negative events, interference, self-monitoring of BG burden, efficacy, social burden, psychological well-being, treatment satisfaction and treatment preference.^25^

HCP experiences and perspectives on insulin dosing prior to the study were collected at baseline, and the acceptability and satisfaction with the MDC was assessed through a study-specific survey at the end of the study. DCESs also kept logs detailing experiences and logistical issues, particularly those related to technology.

### Data analysis

Descriptive statistics were used to report on patient characteristics at baseline and summarize other data (e.g. patient and HCP surveys). Changes in HbA1c and survey scores for diabetes distress and medication system ratings were assessed using paired t-tests. Data are presented as mean (standard deviation [SD]) or frequency (percentage), unless otherwise noted. Data were analyzed using IBM SPSS Statistics (Version 28).

## Results

A total of 42 individuals were eligible and consented to participate in the study. Non-completers were removed (n=3, one death and two medical treatment changes), lost to follow-up (n=2), or withdrew (n=1). Completers (n=36) were female (61.1%), non-Hispanic (94.4%), white (88.9%), married (63.9%) with a mean age of 57.8 years. Average duration of diabetes diagnosis at baseline was 12 years and mean baseline HbA1c was 9.8%. Most patients were previously diagnosed with obesity (88.9%) and/or hypertension (75%). These and other characteristics are presented in *Table 1*.

### Glycaemic outcomes

Mean HbA1c was significantly reduced by an average of 2.1±2.2% from baseline to follow-up (p<0.001), with 31% of participants achieving an HbA1c target value of <7%. These improvements were reflected in a significant reduction in mean FBG from 177.5 ± 82.4 mg/dL at baseline to 144.1 ± 61.2 mg/dL at 3 months (p=0.011), with 69.4% of patients reaching their individualized FBG target. Of those who reached their FBG target at 3 months, the average time to titration target was 28.8 ± 26.1 days. Nine participants had one or more glucose recordings <70 mg/ dL during the course of the study; all cases were considered as mild asymptomatic hypoglycaemia.

### Diabetes distress

At baseline, patients reported clinically significant levels of distress (DDS17 mean scores >2) in the areas of emotional burden (i.e. feeling overwhelmed, frightened or fearful about managing the demands of diabetes over time), regimen distress (i.e. feeling like failing by not managing diabetes well) and interpersonal distress (i.e. feeling like family and friends are not providing sufficient diabetes support).^23,24^ As shown in *Table 2*, diabetes distress was significantly reduced from baseline to follow-up, with DDS17 scores decreasing (or improving) across all scales.

### Patient satisfaction

Mean DMSRQ-SF scores, which represent patients’ comprehensive assessment of their treatment experience, are shown in *Table 3*. The baseline scores reflect the patients’ ratings of their diabetes medication systems prior to the intervention (e.g. medication system they were on before starting basal insulin titration and using MDC). Of note, composite scores significantly improved from baseline to follow-up, as did scores for convenience satisfaction, self-monitoring of blood glucose burden, efficacy, psychological well-being and treatment satisfaction. When specifically considering MDC, patients reported high levels of acceptability and satisfaction with the digital titration app (*Table 4*).

**Table 1:** Patient characteristics

Characteristic	
**Female, n (%)**	22 (61.1)
**Age in years, mean (SD)**	57.8 (11.4)
**Race, n (%)**	
Black	3 (8.3)
White	32 (88.9)
Other	1 (2.8)
**Ethnicity, n (%)**	
Non-Hispanic	34 (94.4)
Unknown	2 (5.6)
**Marital status, n (%)**	
Single	6 (16.7)
Married	23 (63.9)
Divorced	5 (13.9)
Widowed	2 (5.6)
**Education level, n (%)**	
Less than high school diploma	1 (2.8)
High school diploma	10 (27.8)
Post high school training	4 (11.1)
Associate degree/some college	8 (22.2)
Bachelor's degree	11 (30.6)
Graduate degree	2 (5.6)
**Employment, n (%)**	
Unemployed	6 (16.7)
Employed	19 (52.8)
Retired	11 (30.6)
**Insurance, n (%)**	
Medicaid	1 (2.8)
Medicare	17 (47.2)
Commercial/private	17 (47.2)
Unknown	1 (2.8)
**Duration of diabetes diagnosis, mean number of years (SD)**	12 (9.3)
**Body mass index, mean (SD)**	36.5 (8.9)
**Weight (kg), mean (SD)**	107.2 (31.1)
**Blood pressure (mmHg), mean (SD)**	
Systolic	125.7 (23.7)
Diastolic	76.9 (7.4)
**Lipids (mg/dL), mean (SD)**	
Total cholesterol	159.6 (41.6)
LDL	85.4 (34.5)
HDL	43.5 (11.7)
Triglycerides	196.8 (131.6)
**Comorbidities**	
Obesity, n (%)	32 (88.9)
Hypertension, n (%)	27 (75)
Coronary artery disease, n (%)	6 (16.7)
Stroke, n (%)	1 (2.8)
**HbA1c levels (%), mean (SD)**	9.7 (1.6)
**Fasting blood glucose (mg/dL), mean (SD)**	173.8 (78.2)
**Glucose lowering drugs/medications at baseline, n (%)**	
Oral antidiabetic drug	35 (97.2)
Non-insulin injectable	20 (55.6)
Insulin (without titrating)	23 (63.9)
**Insulin prescribed for study, n (%)**	
Insulin glargine 100 IU/mL	22 (61.1)
Insulin glargine 300 IU/mL	14 (38.9)

*HbA1c = glycated haemoglobin type A1c; HDL = high-density lipoprotein; LDL = low-density lipoprotein; SD = standard deviation.*

As for HCPs, prior to the intervention, participating medical providers and DCESs (n=13) had mixed experiences communicating dosing instructions to patients when starting insulin therapy. All reported having some level of concern about their patients understanding of dosing instructions and correctly and safely administering insulin. With regards to titration instructions, 46% of HCPs printed instruction from the Electronic Medical Record (EMR), 31% communicated instructions via intermittent calls with patients, 15% used handwritten instruction and one provider used an online resource. Only one HCP had previous experience with an insulin dosing app. All HCPs agreed that an app like MDC would be beneficial to their patients.

As detailed in *Table 5*, after using MDC with patients during the study, medical HCPs and DCESs reported high levels of satisfaction and positive experiences with MDC. All providers agreed that they thought MDC was a better method for titrating insulin than other methods that they had previously used, and that MDC helped them feel better about providing titration instructions to their patients. HCPs unanimously agreed that they would use MDC again with their patients.

## Discussion

To our knowledge, this is the first study of an insulin titration app utilized by a medical HCP and DCES with a focus on examining the clinical, psychosocial and satisfaction outcomes relevant to diabetes self-management. Our findings showed a meaningful and positive association between the app and integrated team approach on glycaemia and important psychosocial outcomes. This builds on previous reports in support of titration apps in helping patients with T2DM reach glycaemic goals while addressing the growing mismatch between the number of patients and specialized providers who can make dose titrations.^22,26^ In a previous titration study carried out in three diabetes centres, the combination of automated insulin titration guidance with support from HCPs offered superior glycaemic outcomes compared with support from HCPs alone.^26^ The titration app was further examined in another study where nurses played a key role in the study intervention by providing follow-up calls, correcting usage errors, identifying atypical clinical courses and building confidence among users. The intervention was found to be effective in assuring proper use of the titration device and was associated with improvements in HbA1c levels. The investigators maintain that with the nurses’ support, problems were identified earlier and patients were empowered to make their own frequent dosage changes.^22^ In another large population study designed to assess the impact of frequency of the MDC app and titration use, increased user frequency was associated with significantly better FBG target achievement. Users in the high-usage group took the shortest time to achieve FBG targets versus moderate-and low-usage groups (14.9 days versus 25.1 days and 36.8 days, respectively; p≤0.01 for both comparisons).^11^ These results indicate that digital tools to support basal insulin titration could be useful to both people with diabetes and HCPs in diabetes management in terms of achieving improved glycaemic outcomes.

**Table 2:** 3-month reduction in diabetes distress in patients with type 2 diabetes mellitus after initiating basal insulin titration and using a digital titration application to guide insulin dosing

Scale, mean (SD)	Baseline	3 months	p-value
DDS17 total score	1.9 (0.6)	1.5 (0.3)	<0.001
Subscales			
Emotional burden	2.1 (0.8)	1.4 (0.4)	<0.001
Regimen distress	2.2 (0.8)	1.6 (0.4)	<0.001
Interpersonal distress	2 (0.9)	1.6 (0.6)	0.008
Physician distress	1.4 (0.5)	1.2 (0.3)	0.011

*DDS17 possible range: 1–6. n=36.*

*DDS17 = 17-item Diabetes Distress Scale; SD = standard deviation.*

**Table 3:** Assessment of treatment experience by patients with type 2 diabetes mellitus after initiating basal insulin titration and using digital titration app to guide insulin dosing

DMSRQ scale	Baseline, mean (SD)	3 months, mean (SD)	p-value
Composite score (n=34)	69.1 (12.9)	80.4 (10.3)	<0.001
Convenience satisfaction (n=35)	68.5 (28.6)	82.9 (21.2)	0.014
Negative events (n=35)	84 (13.6)	85.6 (10.9)	0.473
Interference	76.9 (30.7)	83.4 (25.8)	0.215
Self-monitoring of blood glucose burden	79.2 (32.5)	97.2 (16.7)	<0.001
Efficacy (n=35)	46.2 (19.5)	63.6 (24.6)	<0.001
Social burden	83.3 (26.7)	84.7 (30.0)	0.773
Psychological well-being	63.5 (22.2)	76.7 (18.7)	0.002
Treatment satisfaction	55.6 (24.8)	81 (17.1)	<.001

*Possible range for DMSRQ scale scores: 0–100. (n=36, except where noted).*

*DMSRQ = Diabetes Medication Self-Rating Questionnaire; SD = standard deviation.*

**Table 4:** Patient ratings of My Dose Coach digital titration application

Survey item	Mean score (out of 5)
The app was easy to learn	4.7
The app is simple to use	4.7
The app is simple and easy to understand	4.7
I like using the app	4.7
I believe I could quickly be able to use this app to adjust my insulin	4.6
I think the app can provide better support for insulin dosing than a handwritten or printed dose scale	4.6
I feel safe getting insulin dose guidance through the app	4.6
I feel comfortable communicating with my clinician/educator using the app	4.7
The app is an acceptable way to receive insulin dosing instructions	4.7
I would use the app again	4.8
Overall, I am satisfied with this app	4.8

*n=36.*

*app = application.*

**Table 5:** Healthcare provider ratings of My Dose Coach digital titration application

Survey item	Average score (out of 5)
The system (app and clinical portal) was simple to use	4.4
Setting up a patient's dosing algorithm in the clinical portal was easy to do	4.3
It was easy to teach patients how to use the app	4.3
It would be useful if blood glucose and/or insulin dose data could be automatically uploaded to the app (versus manually entered by patients)	4.4
I trusted the dosing instructions that the app provided to my patients	4.7
I felt safe with my patients using the app	4.7
The app was better for titrating insulin than other methods (phone calls, paper scale)	4.8
I liked using the system to provide insulin dosing instructions to my patients	4.8
The system helped me feel better about providing titration instructions to my patients	4.8
I would use the app again with my patients	4.9
Overall, I was satisfied with system	4.8

*n=12.*

*app = application.*

A strength of our study is that it sheds light on the impact of digital tools on important psychosocial outcomes. People with T2DM have been reported to experience diabetes distress, defined as a group of responses to living with diabetes, associated with treatment and self-management demands.^27^ Diabetes distress has been associated with poor clinical outcomes and treatment adherence, and the inability to reach glycaemic treatment goals. In a study examining the relationship between diabetes distress and glycaemic control following participation in DSMES, researchers found a reduction in diabetes distress that was associated with a 0.25% reduction in HbA1c over a 6-and 12-month period.^28^ The ADA now recommends HCPs refer patients to DSMES to focus on self-care needs, including concerns about their ability to maintain a diabetes treatment regimen.^27^ Recognizing the benefits of DSMES in insulin management, we presume that it contributed to the reduction in diabetes distress experienced by our study participants, an important factor for effective ongoing self-care. Critical to the success of a collaborative team approach in introducing a new clinical tool is provider satisfaction. In addition to high satisfaction of MDC in patients, both medical HCPs and DCESs surveyed regarded the tool to be a valuable asset in titration management. They found MDC to be safe, simple to use (including setting up algorithms), easy to teach and better than currently-available methods, and would use it again. In addition, DCES anecdotally reported that patients not only liked using the app but had better adherence to insulin dosing when using it.

Although this study has found that the MDC app can afford ways to address the daily challenges of patient insulin titration and has introduced the DCES as another HCP who can properly train and manage the titration process for improvements in glycaemia, we recognize that there are several limitations that must be taken into consideration when interpreting findings. First and foremost, the study was implemented during the coronavirus disease-19 (COVID-19) pandemic. When in-person visits were limited by the pandemic, patients had little choice in accepting virtual visits and its associated new challenges. For example, it limited opportunities for in-person contact to introduce and invite participation in the intervention. While this may have influenced recruitment, DCES reported that patients liked using the app and that only one patient was reported to need a return visit for additional training. Recruitment was challenging despite providers' interest in study participation. Staffing shortages and work overload during the COVID-19 pandemic limited their time and ability to recruit patients, which resulted in a smaller sampler size than originally planned, thus limiting the strength of the findings and warrant caution when interpreting results. Given the small sample, we did not examine the association of patient characteristics with glycaemic goals, and this would need to be considered in future investigations that include a large, diverse population.

In addition, while our sample was diverse in several ways, including in education, insurance coverage and residence within our geographic region, findings may be limited in their generalizability to other populations not fully represented in our sample. Despite these limitations, evidence summarized here suggests that a collaborative team that includes a DCES who is trained to provide an active role in therapeutic management can work together to provide patient care and education with automated titration guidance technology. The model offers another solution to streamline insulin dosage adjustments to achieve specific clinical and self-management goals with high expectations for long-term benefits.

## Conclusions

Frequent and proper insulin titration is essential for effective insulin therapy; however, adequate patient training and self-management support is often limited in current healthcare settings. Our study offers insights into factors found beneficial for successful titration and integration of an interconnected insulin dosing system into real-l ife practice and sets the stage for future investigation.
